# Gene Expression Profiling in a Mouse Model Identifies Fetal Liver- and Placenta-Derived Potential Biomarkers for Down Syndrome Screening

**DOI:** 10.1371/journal.pone.0018866

**Published:** 2011-04-14

**Authors:** Jeroen L. A. Pennings, Wendy Rodenburg, Sandra Imholz, Maria P. H. Koster, Conny T. M. van Oostrom, Timo M. Breit, Peter C. J. I. Schielen, Annemieke de Vries

**Affiliations:** 1 Laboratory for Health Protection Research (GBO), National Institute for Public Health and the Environment (RIVM), Bilthoven, The Netherlands; 2 Laboratory for Infectious Diseases and Perinatal Screening (LIS), National Institute for Public Health and the Environment (RIVM), Bilthoven, The Netherlands; 3 MicroArray Department (MAD), University of Amsterdam (UvA), Amsterdam, The Netherlands; University Hospital Vall d'Hebron, Spain

## Abstract

**Background:**

As a first step to identify novel potential biomarkers for prenatal Down Syndrome screening, we analyzed gene expression in embryos of wild type mice and the Down Syndrome model Ts1Cje. Since current Down Syndrome screening markers are derived from placenta and fetal liver, these tissues were chosen as target.

**Methodology/Principal Findings:**

Placenta and fetal liver at 15.5 days gestation were analyzed by microarray profiling. We confirmed increased expression of genes located at the trisomic chromosomal region. Overall, between the two genotypes more differentially expressed genes were found in fetal liver than in placenta. Furthermore, the fetal liver data are in line with the hematological aberrations found in humans with Down Syndrome as well as Ts1Cje mice. Together, we found 25 targets that are predicted (by Gene Ontology, UniProt, or the Human Plasma Proteome project) to be detectable in human serum.

**Conclusions/Significance:**

Fetal liver might harbor more promising targets for Down Syndrome screening studies. We expect these new targets will help focus further experimental studies on identifying and validating human maternal serum biomarkers for Down Syndrome screening.

## Introduction

Prenatal screening for Down Syndrome (DS) has been routinely available for two decades. Typically, such screening procedures consist of a risk calculation based on maternal age, nucal translucency and serum biomarker measurements, after which women with a high predicted risk can opt for invasive testing such as amniocentesis or chorionic villus sampling. Initially, the most commonly used method for risk calculation was the second trimester triple test, which combines serum levels for alpha-fetoprotein (AFP), unconjugated estriol (uE3), and the free β subunit of human chorion gonadotrophin (fβ-hCG) with maternal age [1], [2]. Currently, many countries including the Netherlands, have replaced this by the first trimester combined test, which is based on fβ-hCG and pregnancy-associated plasma protein A (PAPP-A) serum concentrations, ultrasound nuchal translucency (NT) measurements and maternal age [3]. This latter test has a Detection Rate (DR) of 75–85% at a 5% false positive rate (FPR) [4]–[6]. Although the reliability of the first trimester combined test is better than the triple test, both the DR and the FPR are still in need for improvement, and a lot of international effort has been put in improving both kinds of prenatal tests.

A promising approach to improve DS screening is by adding multiple biochemical markers to the serum analysis. By means of innovative proteomics, genomics, and bioinformatics approaches, novel discriminative markers can be identified that, when added to the current serum assays, can improve the DR and FPR [7]–[12].

Serum markers used in these two routinely used screening tests essentially originate from two tissues, namely fetal liver (AFP) and the placenta (fβ-hCG, PAPP-A), whereas the non-protein serum biomarker uE3 is produced by the placenta from its precursor dehydroepiandrosterone sulfate produced by the fetal liver and adrenal glands. We therefore hypothesize that placenta and fetal liver harbor additional biomarkers suitable for improving DS screening, and have set up a research strategy to identify them. Availability of fetal human material for DS cases or controls is limited and therefore existing human studies are restricted to placenta or cultured trophoblasts [13]–[16]. Additionally, when human material is available, genomics and proteomics studies are inevitably complicated by sources of variation from maternal, fetal, and clinical origin.

A possibility to overcome such limitations is the use of inbred animal models. For ethical and practical reasons, mouse models are preferable for such studies, and fortunately several mouse models are available mimicking human Down syndrome [17]–[24]. In this study we used the Ts1Cje mouse strain [21], which contains a segmental trisomy of mouse chromosome 16 (Mmu16) distal of the *Sod1* gene, including a region orthologous to the region of human chromosome 21 commonly associated with Down Syndrome: the “Down Syndrome critical region” [21]. We selected a mouse model in which the Mmu16 trisomic region extends beyond the DSCR, as comparative genetic studies [25], [26] have indicated that trisomy for only the DSCR is not sufficient for a complete DS phenotype. Ts1Cje mice have been shown to display a recognizable DS phenotype which consists of craniofacial malformations including a smaller cerebellum volume, as well as learning and behavioral abnormalities [17], [21], [27].

In this study, fetuses were obtained from wild type mothers bred with either wild type or Ts1Cje males. Gene expression profiles in fetal liver and placenta of wild type and Ts1Cje fetuses were compared and for differentially expressed genes it was examined if they code for blood detectable proteins and/or are involved in clinically involved processes. With this strategy, we have identified a number of targets with potential for further studies ultimately aimed at biomarker application in human prenatal DS screening.

## Materials and Methods

### Ethics statement

This study was agreed upon by the Animal Experimentation Ethical Committee of our institute under permit number 200900176. Animal handling in this study was carried out in accordance with relevant Dutch national legislation, including the 1997 Dutch Act on Animal Experimentation.

### Animal studies

The trisomic B6EiC3Sn-Ts(16C-tel)1Cje1 mice, also named Ts1Cje, contain an additional copy of distal chromosome 16 [21]. Trisomic B6EiC3Sn-Ts(16C-tel)1Cje1 mice (genotype Ts/+) and wild type hybrid background B6EiC3SnF1/J mice (genotype +/+) were purchased from the Jackson laboratory (Bar Harbor, ME, USA).

To obtain Ts1Cje and wild type fetuses for RNA isolation, male +/+ mice (control group) or male Ts/+ mice (Down group) were bred with female breeding mice of the C3H/HeNHsd strain (Harlan, Horst, the Netherlands) at 8–10 weeks of age. After mating, females were separated and pregnant females were identified through scoring of vaginal plugs (embryonic time point E0.5 in days). Females were sacrificed on E15.5 using CO_2_/O_2_. From pregnant mice all embryos were collected and every single embryo was processed further. Placenta and fetal liver were collected for RNA extraction and paws were collected for DNA extraction and genotyping. All tissues were immediately frozen in liquid nitrogen and stored at −80°C until further processing.

### DNA extraction, embryo genotyping and sex determination

Genomic DNA was extracted from embryo paws. Genotyping and sex determination on mice embryos were both performed by multiplex PCR using primer sequences given in Supporting Dataset S1. Each PCR contained 5 µl 2× Hotstar Master Mix (Qiagen), 0.5 µM of each primer and 10–50 ng genomic DNA, in a total volume of 10 µl. PCR reactions were carried out in a Perkin-Elmer 9600 thermal cycler under the following conditions: 95°C for 15 min; 30 cycles of 94°C for 30 sec, 55°C for 30 sec, 72°C for 1 min; followed by 72°C for 10 min.

### RNA isolation, yield and quality

RNA was extracted from placenta and fetal liver using the miRNeasy kit (Qiagen). RNA concentrations were measured using a NanoDrop spectrophotometer (NanoDrop Technologies, Wilmington, DE, USA). The integrity of the RNA samples was determined with the BioAnalyzer (Agilent Technolgies, Amstelveen, The Netherlands) using the RNA nano 6000 kit (Agilent Technologies) yielding RIN-values ≥9.6. For placenta and fetal liver, microarray analysis was carried out using RNA samples of 24 individual embryos, *i.e.* six male and six female embryos from both genotypes.

### Amplification and labeling protocol

Per sample, 500 ng total RNA was amplified according to the Agilent QuickAmp kit manual (Agilent technologies). Amino-allyl modified nucleotides were incorporated during the aRNA synthesis (2.5 mM rGAU (GE Healthcare), 0.75 mM rCTP (GE Healthcare), 0.75 mM AA-rCTP (TriLink Biotechnologies). Synthesized aRNA was purified with the E.Z.N.A. MicroElute RNA Clean Up Kit (Omega Bio-Tek). Test samples were labeled with Cy3 and a Reference sample (made by pooling equimolar amounts of RNA from Test samples) was labeled with Cy5. Next, 5 µg of aRNA was dried down and dissolved in 50 mM carbonate buffer pH 8.5. Individual vials of Cy3/Cy5 from the mono-reactive dye packs (GE Healthcare) were dissolved in 200 µl DMSO. To each sample, 10 µl of the appropriate CyDye dissolved in DMSO was added and the mixture was incubated for 1 h. Reactions were quenched with the addition of 5 µl 4 M hydroxylamine (Sigma-Aldrich). The labeled aRNA was purified with the E.Z.N.A. MicroElute RNA Clean Up Kit. The yields of aRNA and CyDye incorporation were measured on the NanoDrop ND-1000.

### Microarray hybridization, scanning & data processing

Each hybridization mixture consisted of 1.1 µg Test (Cy3) and 1.1 µg Reference (Cy5) sample. Samples were dried and 1.98 µl of the appropriate sample tracking control (STC, Roche Nimblegen) was added. The hybridization cocktail was made according to the manufacturer's instructions (Nimblegen Arrays User's Guide – Gene Expression Arrays Version 5.0, Roche Nimblegen). From this mix, 5.22 µl was added to each sample. The samples were incubated for 5 min at 95°C and 5 min at 42°C prior to loading. Hybridization samples were loaded onto a 12×135 k *Mus musculus* microarray (Catalog no. 05543797001, Design 090901 MM9 EXP HX12) containing probes for 44,170 genes with 3 spots per target probe. Microarrays were hybridized for 20 hours at 42°C with the NimbleGen Hybridization System 4 (Roche Nimblegen). Afterwards, the slides were washed according to the Nimblegen Arrays User's Guide – Gene Expression Arrays Version 5.0 and scanned in an ozone-free room with a Agilent DNA microarray scanner G2565CA (Agilent Technologies). Feature extraction was performed with NimbleScan v2.5 (Roche Nimblegen). Each microarray corresponded to labeled RNA from one individual embryo.

### Data analysis

Complete raw and normalized microarray data and their MIAME compliant metadata have been deposited at GEO (www.ncbi.nlm.nih.gov/geo) under accession number GSE24272.

Raw microarray signal data were normalized in R (www.r-project.org), using a four step approach [28]: (1) natural log-transformation, (2) quantile normalization of all scans, (3) correcting the sample spot signal for the corresponding reference spot signal and (4) averaging data from replicate probe spots. Normalized data for the resulting 44170 probes were further analyzed in R and Microsoft Excel.

For both placenta and liver, gene expression differences between either sex or genotype were compared with an ANOVA. Obtained p-values were corrected for multiple testing by calculating the false discovery rate (FDR) according to Benjamini and Hochberg [29]. Probes with a False Discovery Rate (FDR)<0.05 were considered significant. When multiple probes corresponding to the same gene were significant, their data were averaged to remove redundancy in further analysis. Probes with significant expression differences between male and female embryos were excluded from the analysis on genotype differences.

Hierarchical clustering analysis was performed using GeneMaths XT (Applied Maths, St-Martens-Latem, Belgium) using Euclidean distance and Ward linkage. Functional Annotation and Gene Ontology (GO) term enrichment were examined with the DAVID Bioinformatics Resource (http://david.abcc.ncifcrf.gov) [30]. Enrichment for tissue-specific or literature-based functional gene sets was determined in R using an in-house developed algorithm based on the DAVID methodology. Tissue- or lineage-specific gene sets were obtained from data downloaded from the BioGPS website (http://biogps.gnf.org) [31], [32] as well as other relevant literature sets [33]–[35].

Groupwise regulation of Gene Ontology categories and above-mentioned custom gene sets were determined by Gene Set Enrichment Analysis (GSEA) [35] using default analysis parameters. Gene sets were considered regulated if the GSEA p-value was<0.05 and the FDR was <0.10.

To determine which genes code for proteins detectable in human serum, we determined which proteins are annotated in Gene Ontology as extracellular, in UniProt as secreted, or have been experimentally detected in the Human Plasma Proteome project [36].

### Quantitative RT-PCR

Microarray results were for a subset of genes verified by quantitative RT-PCR analysis on RNA from 12 Ts1Cje versus 12 WT samples. For this, all reagents, methods and equipment were obtained from Applied Biosystems. TaqMan gene expression assays used are given in Supplementary Dataset S2. Assays for *Hprt* and *Polr2a* were custom-made and included as endogenous controls. After RNA samples were reverse transcribed to cDNA, qPCR was performed on 125 ng of cDNA using the 7500 Fast real-time PCR system. Threshold cycles were automatically derived from the amplification plots constructed of the ROX-normalized fluorescence signals by 7500 Fast system SDS software v1.3. The average of the *Hprt* and *Polr2a* level per cDNA sample was used to normalize the expression of the other genes. Relative quantification of the mRNA copies in the Ts1Cje samples compared to that of the WT samples was performed by the comparative threshold cycle method using Microsoft Excel.

## Results

### Genotype confirmation

**Figure 1:**
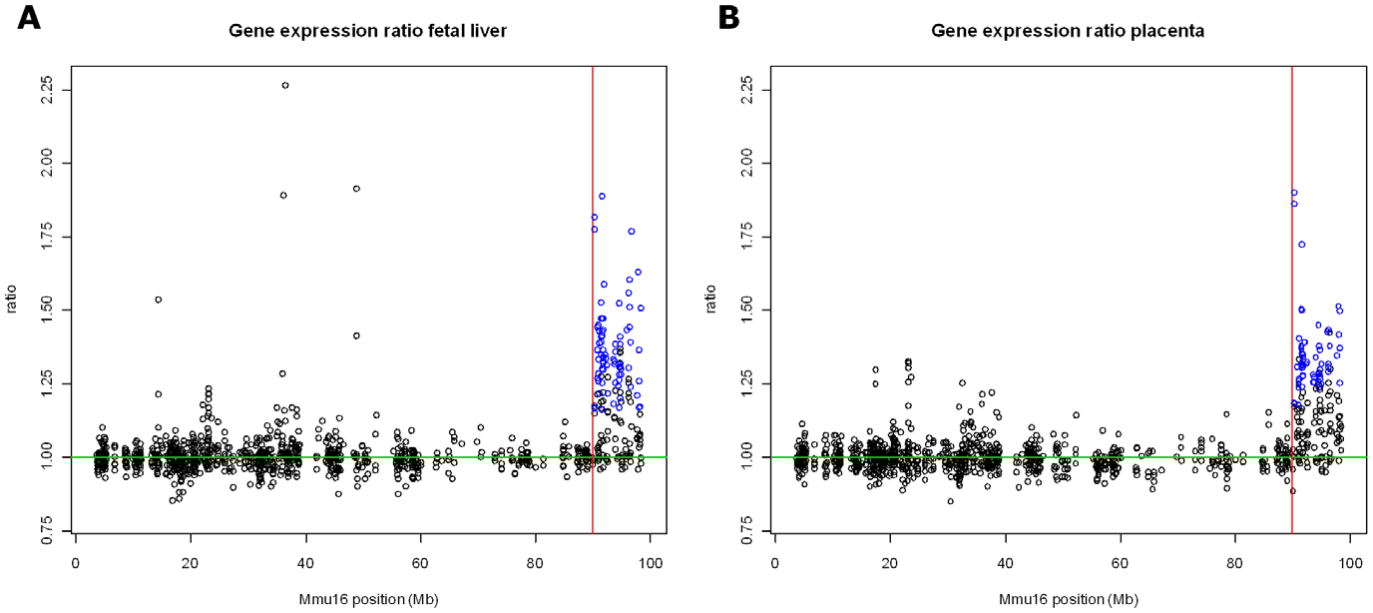
Chromosome plot of Mmu16 with gene expression ratios between Ts1Cje and wild type mice. Significant genes (FDR 5%) are indicated in blue. The border of the trisomic locus is indicated with a vertical red line.

To validate the use of a mouse model for DS in a transcriptomics study, we first compared the expression ratio between Ts1Cje and WT embryos for genes located on chromosome Mmu16. Plotting the gene expression ratio against the chromosomal position (Fig. 1) reveals an increased expression for genes in the segmental trisomic locus in both fetal liver and placenta.

### Sex-specific gene regulation

Comparing differences in expression levels between male and female embryos of either genotype revealed 31 significant probes (12 genes) in fetal liver and 25 significant probes (11 genes) in placenta. When combined, this resulted in 16 genes for which corresponding probes were excluded from the analysis of genotype differences. Briefly, 7 genes were male-specific genes and 9 female-specific, and only 3 out of these 16 genes were not located on either of the sex chromosomes.

### Genotype-specific gene expression in fetal liver

**Figure 2:**
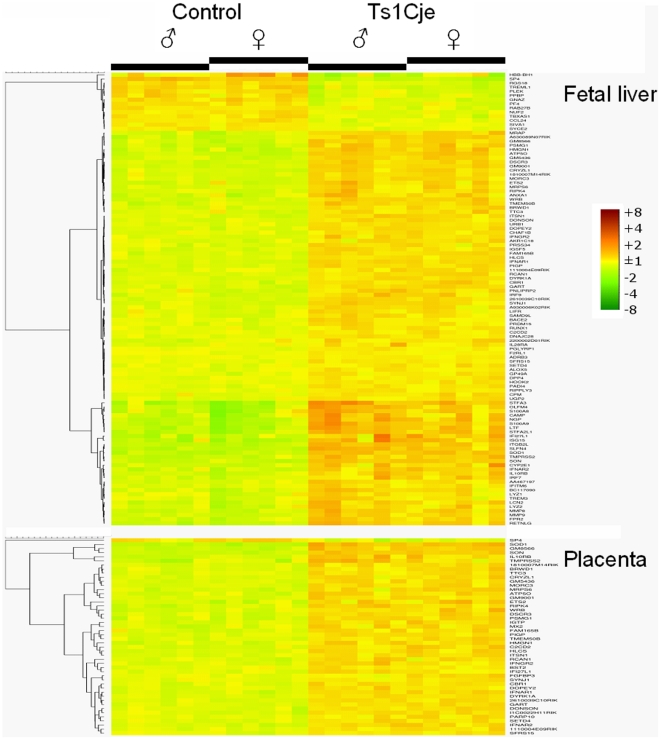
Heatmap for fetal liver and placenta.

For fetal liver, we found significant genotype-related expression changes for 152 probes, corresponding to 109 genes (Supporting Dataset S3). As indicated in the heatmap in Fig. 2, the majority of these (95 genes) are induced in fetal livers of Ts1Cje mice of either sex, with the other 14 being suppressed. Of these, 51 are mapped on the corresponding trisomic locus (Fig. 1).

Functional overrepresentation analysis shows that among the genes with differential expression, there is enrichment for genes involved in immunology and hematopoiesis, including such genes as the calgranulin A and B subunits (*S100a8*, *S100a9*), lactotransferrin (*Ltf*), matrix metallopeptidase 8 and 9 (*Mmp8*, *Mmp9*), platelet factor 4 (*Pf4*), pleckstrin (*Plek*), pro-platelet basic protein (*Ppbp*), and the gene for the zeta hemoglobin chain (*Hbb-bh1*). This enrichment is especially strong among genes that are induced in Ts1Cje mice but are not located on the trisomic locus. Among these non-locus genes, significantly enriched gene sets are mainly associated with the myeloid and neutrophil lineages. Among genes with lower expression in Ts1Cje fetal liver, there is significant overrepresentation of genes associated with or expressed in the platelet lineage.

Threshold-free pathway analysis using GSEA indicated that Ts1Cje mice have increased pathway activity in several GO-terms related to hematopoiesis (*e.g.* leukocyte differentiation, response to virus, response to biotic stimulus) and metabolism (alcohol metabolic process, glycerolipid metabolism, glycolysis and gluconeogenesis). Several custom gene sets related to interferon response, myeloid lineage, and (neutrophilic) granulocytes were induced in Ts1Cje whereas the opposite effect was found for terms related to platelets and (B- and T-) lymphocytes. Excluding the trisomic locus from the data used in the analysis did not significantly change these findings.

Among the 95 genes with significant expression differences in liver, there are 24 that encode for proteins potentially detectable in human blood (Supporting Dataset S3, Table 1). Of these, only 7 are located at the trisomic locus. Many of the other blood-detectable proteins are associated with either neutrophils or platelets.

**Table 1:** Potential blood-detectable biomarkers regulated in fetal liver or placenta.

Gene symbol	Ratio fetal liver	Ratio placenta	Chromosome
*Induced in Ts1Cje mice (at DS locus)*			
C2cd2	1.173	1.370	16
Dyrk1a	1.290	1.282	16
Ifnar2	1.525	1.375	16
Morc3	1.372	1.340	16
Sfrs15	1.166	1.185	16
Sod1	1.796	1.881	16
Tmprss2	1.629	1.514	16
*Induced in Ts1Cje mice (outside DS locus)*			
Camp	1.724	NS	9
Dpp4	1.178	NS	2
Isg15	1.947	NS	4
Lcn2	1.486	NS	2
Lifr	1.238	NS	15
Ltf	1.981	NS	9
Mmp8	1.532	NS	9
Mmp9	1.469	NS	2
Olfm4	1.895	NS	14
Pglyrp1	1.124	NS	7
Pnliprp2	1.345	NS	19
S100a8	1.769	NS	3
S100a9	1.922	NS	3
Fgfbp3	NS	1.178	19
*Suppressed in Ts1Cje mice*			
Ccl24	−1.221	NS	5
Pf4	−1.259	NS	5
Plek	−1.346	NS	11
Ppbp	−1.336	NS	5

NS: not significant.

### Genotype-specific gene expression in placenta

Gene expression profiling for placental RNA revealed 75 probes with statistical significance, corresponding to 48 genes (Fig. 2, Supporting Dataset S3). For this tissue, induced expression in the Ts1Cje placentas was found for 47 genes, 41 of which are located on the trisomic locus (Fig. 1). Only one gene (*Sp4*) was suppressed in Ts1Cje mice. No significant overrepresentation for pathways or other gene sets was found among placenta-regulated genes.

GSEA found no significant pathway-level effect among GO-terms, and among the custom gene sets included, significant scores were only observed for leukocytes, especially neutrophils. However, significance was less pronounced than in fetal liver and excluding the trisomic locus further reduced the extent of this effect.

Among the genes regulated in placenta, 8 have human homologs that are blood- detectable at the protein level (Supporting Dataset S3, Table 1). With the exception of *Fgfbp3*, these are the same 7 markers located on the trisomic locus as for the fetal liver.

### Overlap

The overlap between regulated genes in placenta and fetal liver comprises 42 genes. Of these, 40 are located in the trisomic region, of which 7 genes encode for potentially blood-detectable proteins (*C2cd2*, *Dyrk1a*, *Ifnar2*, *Morc3*, *Sfrs15*, *Sod1*, *Tmprss2*). Of the two genes that are not located on the trisomic locus, *Ifi27l1* was increased and *Sp4* had decreased expression in Ts1Cje mice compared to WT mice. For neither of these two genes there is evidence for protein detectability in human blood.

### Quantitative RT-PCR verification

For two genes with increased expression in Ts1Cje placenta as well as fetal liver (*Sod1* and *Dyrk1a*) and four with differential expression in Ts1Cje fetal liver (*Pf4*, *Ppbp*, *S100a8*, *S100a9*) we performed quantitative RT-PCR (Supporting Dataset S2). For all of these six genes, we confirmed their differential expression as well as their significance at p<0.05. The direction of change was in agreement for all assays. Expression changes measured by PCR were comparable to those measured by microarray, with the median difference between microarray versus RT-PCR ratios being 11% (Supporting Dataset S2).

## Discussion

Gene expression profiling in animal models has been previously successfully applied to gain insight and discover novel protein biomarkers for detection of human diseases [37]–[39]. For DS, several mouse models have been developed to study the effect of trisomy in single or multiple genes on DS phenotype and development (reviewed in [17], [18]). Of these models, the Ts1Cje and Ts65Dn mice have so far been used most for gene expression analysis on brain tissue [40]–[44] and to a lesser extent on other adult tissues [45], [46]. In this study, we describe for the first time gene expression analysis on fetal tissue of DS model mice with the ultimate goal to identify potential biomarkers applicable for prenatal serum screening. Although over a dozen mouse models for DS have been described in the literature, not all of these are equally useful for DS screening biomarker discovery by gene expression comparisons during fetal development. In mouse models with trisomies for only single genes, the phenotype is less pronounced whereas, on the other hand, mouse models with trisomies for larger segments or even an entire chromosome tend to suffer from male infertility or fetal death. The Ts1Cje mouse shows a recognizable DS phenotype while still allowing for breeding, and was consequently chosen as a model in this study. Gene expression data were compared in fetal liver and placenta between Ts1Cje and wild type embryos of both sexes at gestational age 15.5 days. This age corresponds to the developmental phase at the end of the first trimester in humans (Carnegie stage 22). Because the combined first trimester test is carried out at this time point, the corresponding mouse gestational age was chosen as the optimal time point for DS biomarker discovery.

As expected, gene expression data showed an increased expression of genes located in the trisomic locus (Fig. 1). This is in agreement with the gene dosage effect described earlier in human DS as well as mouse models [41]–[43], [45]–[47]. In addition, in each tissue we observed sex-specific expression differences for some genes, most of which were located on either the X or Y chromosome. However, for eventual human implementation in a pregnancy screening program, markers should not show sex-specific differences. Firstly, because the accuracy of the screening program (DR and FPR) will benefit most from DS-markers that are applicable to both male and female embryos. Additionally, if sex-specific markers were to be included in a blood test, this would complicate the counseling to pregnant women. Therefore, in this study, these sex-specific markers were primarily identified in order to be excluded from the main analysis.

Comparing fetal liver RNA from Ts1Cje with wild type embryos, we found differential expression for 109 genes, of which slightly more than half (58 genes) were outside the trisomic locus. Remarkably, functional enrichment is stronger among these 58 genes than among the 109 genes as a whole. This indicates that although a large fraction of the differentially expressed genes are located in the same chromosomal regions, the main functional effect is due to genes from multiple other chromosomes. Functional enrichment analysis provided evidence that in Ts1Cje fetal liver there was an increased expression in immune- en hematopoiesis-related genes, more specifically of those expressed in the (early) myeloid and neutrophil lineages, with a concurrent lower expression of platelet-associated genes. As the fetal liver represents the major organ of hematopoietic development during the fetal period in mice as well as in humans, these findings indicate a disturbed hematopoiesis in fetal Ts1Cje mice. Humans with DS also suffer from various hematological abnormalities, including thrombocytopenia, neutrophilia, and macrocytosis. For example, around 10% of human DS newborns have transient megakaryoblastic leukemia. This disease is unique to DS and constitutes proliferation of immature megakaryoblasts. In most cases, this disorder resolves later in life, but in 20–30% it develops into acute megakaryocytic leukemia [48]. Carmichael *et al.* described that although Ts1Cje mice do not develop either of the two leukemic disorders, fetal liver hematopoiesis is nevertheless perturbed in Ts1Cje mice [49], with the main defects in the hematopoietic stem cell and myeloid progenitor cell compartments [49]. Their findings are reflected in the gene expression data described in this study and the functional parallels between murine and human fetal hematopoiesis abnormalities indicates that these markers can be prioritized with regard to human follow-up studies.

Placental gene expression data show differential expression for 48 genes. Most of these genes can be ascribed to gene dosage effects of the trisomic locus at Mmu16. Functionally, there is no significant overrepresentation of functional categories among the differentially expressed genes, although GSEA indicates increased levels of neutrophil-associated genes. In light of the data found for fetal liver, this probably does not indicate an effect occurring primarily in the placenta, but rather results from an increased neutrophil count throughout the embryos as a whole, being detected in the placenta as this tissue is rich in blood vessels. Increased levels in the placenta could lead to increased fetal-maternal exchange of the associated proteins, which could be detected in a screening assay provided they exceed the background variation in maternal blood.

We detected only a small number of non-trisomic genes differentially regulated in placenta. Furthermore, we could not detect a significant effect in *Pappa* (*Papp-a*, ratio −1.01, p = 0.627) or in other placental genes that have been described as biomarkers for DS (e.g. *Adam12*: ratio = −1.01, p = 0.397; *Inha*: ratio = −1.00, p = 0.271; *Pgf* (*Plgf*): ratio = 1.02, p = 0.648). It should be noted here that mice lack the genes corresponding to β-hCG or PP13 and therefore these particular comparisons cannot be made. Although it should not be taken for granted that Ts1Cje mice are a suitable model for serum biomarker discovery regarding human DS screening, our finding does not stand on its own. Comparable studies using human placental(-) samples also could not verify gene expression changes for known screening biomarkers [13]–[16]. A possible explanation might be that the regulation of some biomarker serum levels does not primarily occur at the gene expression levels but at one of several post-transcriptional stages. Alternatively, it needs to be considered that placenta is a relatively heterogeneous tissue, consisting of various cell types from the embryo as well as the mother. Therefore, only a small percentage of placental cells might produce the specific serum biomarkers, so that gene expression measurement in placenta as a whole will give attenuated responses that are statistically more difficult to detect.

A comparison between placenta and fetal liver showed there were two genes (*Ifi27l1* and *Sp4*) regulated in a similar manner in both tissues, but not located on the trisomic locus or sex chromosomes. Concerning the latter, it must be noted that Laffaire *et al.* recently described high resolution comparative genomic hybridization data that show how the translocation of the distal part of Mmu16 to the telomeric part of Mmu12 in Ts1Cje mice results in a deletion of a 2 Mb part containing 5 genes (*Dnahc11*, *Sp4*, *Sp8*, *Abcb5* and *Itgb8*) [43]. Because Ts1Cje mice are monosomic for this fragment, the lower *Sp4* expression found in both fetal liver and placenta of Ts1Cje mice can therefore probably be also attributed to a gene dosage effect. When gene expression data for the other genes in this monosomic locus were compared, we found that they are not expressed at detectable levels in either fetal liver or placenta, which explains why there is no differential expression found for these genes. *Ifi27l1* is also located on chromosome 12, but in an unaffected region, and therefore its differential expression is presumably independent from gene dosage effects. However, because *Ifi27l1* codes for a protein that is not located extracellularly, it is not likely to be useful as a serum biomarker.

An overall comparison between the data obtained for fetal liver and placenta found that more differentially expressed genes were found in fetal liver than in placenta, and that the findings in fetal liver can also be better matched to the pathological features observed in mice and humans. Although current searches for new serum biomarkers that can improve the DS screening accuracy are very much focused on the role of the placenta [50], this study suggests that fetal liver might nevertheless still be of sufficient value in this respect to warrant further studies. Indeed, of the fetal liver hematopoiesis-associated genes, 4 have been suggested as potential biomarkers in a literature data mining study from our laboratory [10]. Two of these (*Pf4*, *Ppbp*) were decreased, whereas both *S100a8* and *S100a9* were increased in Ts1Cje mice. We are aware that any maternal serum level changes in one of these individual markers might originate in changes in the maternal immune or hematopoietic system. Therefore, we put forward that follow-up studies should first determine background levels and variation in maternal blood, and additionally should not focus on single proteins, but rather on concurrent changes in these four markers [38].

In addition to these affected genes shared between both tissues, we also found 7 trisomic genes that are significantly regulated in both mouse tissues and potentially detectable in human serum. These include *Sod1* and *Dyrk1a*, which have been described to be associated with DS pathogenesis in the literature [17], [18], [51], [52]. The corresponding proteins for these genes might therefore also provide potential targets for further study in human maternal serum. Measurement of biomarkers originating in the DS trisomic genotype can have an extra benefit compared to other potential markers. DS screening biomarkers that are currently used, or considered as candidates, are not located on human chromosome 21 and are also predictive for other aneuploidies such as Edwards syndrome (trisomy 18) and Patau syndrome (trisomy 13). It can be expected that markers located on the DS region are not only informative to distinguish DS from normal pregnancies, but also to differentiate between DS from other kinds of fetal chromosomal aberrations. This added information might be an additional reason to include such markers in as screening test.

In DS screening research, the use of omics methods has in recent years contributed to the identification of several markers that have the potential to improve DS screening accuracy [7]–[12]. However, human cohort serum studies are restricted by limited sample availability, large clinical variations, and additionally substantial costs in terms of laboratory equipment and reagents. In several other research fields, animal models are used to partially overcome such limitations. In this study, we report on the first use of a mouse model to identify a set of potential targets aimed at supporting human biomarker studies by providing a more focused starting position. Altogether, based on our gene expression analysis we describe 25 targets for DS screening studies (Table 1), 6 of which (*Pf4*, *Ppbp*, *S100a8*, *S100a9*, *Sod1*, *Dyrk1a*) have been described earlier to be associated with DS [10], [17], [18], [51], [52]. For these latter 6 targets, we confirmed their differential expression by quantitative RT-PCR (Supporting Dataset S2). Evidently, since we identified these new targets in a gene expression study, it still needs to be determined if the changes in RNA levels result in changes at the serum protein level that exceed maternal background levels at a time point suitable for screening. To this end, identification and validation of these targets at the protein level in human serum from pregnant women carrying normal and DS fetuses still has to be performed. As there is ongoing interest [53]–[55] in how determining fetal RNA and/or (methylated) DNA in maternal plasma can detect DS or other aneuploidies, in such further human studies it might be worth while to find out if such methodology is applicable to these 25 or even other regulated genes (Supporting Dataset S3). However, this study, to our opinion, narrows down the list of potential serum targets to be studied in subsequent case-control biomarker discovery experiments, which is extremely important given the enormous labor and financial efforts associated with the identification and validation of potential biomarkers. In this light, it can also be noted that in order to efficiently perform further human case-control experiments on identified targets, the serum measurements should preferentially be performed by means of a multiplexed assay to keep the workflow and the amount of required serum within reasonable limits. In further, more focused studies, assessing the feasibility of determining serum levels of these 6 targets combined with the currently used markers in a multiplexed assay format will therefore have high priority.

## Supporting Information

Dataset S1 **Primers used for sex and genotype determination (Microsoft Word document).**
(DOC)

Dataset S2 **QPCR validation (Microsoft Excel document).**
(XLS)

Dataset S3 **Regulated genes for fetal liver and placenta (Microsoft Excel document).**
(XLS)
