# Supplementary material for: Gene Expression Profiling in a Mouse Model Identifies Fetal Liver- and Placenta-Derived Potential Biomarkers for Down Syndrome Screening
Source: PLoS One. 2011 Apr 14;6(4):e18866. doi: 10.1371/journal.pone.0018866 (PMC3077415; doi:10.1371/journal.pone.0018866)
Supplement: Dataset S1 — Primers used for sex and genotype determination (Microsoft Word document). (DOC) [file pone.0018866.s001.doc]

**Supplemental Table 1**

Primers used for genotyping

(from: Sago et al, Proc Natl Acad Sci U S A 1998; 95:6256-61)

| Primer Name | Sequence (5’-3’) | Nt | Product | Ta |
| --- | --- | --- | --- | --- |
| Neo3 | CTC ACC TTG CTC CTG CCG AG | 20 | 150 bp | 55 °C |
| Neo4 | CTG ATG CTC TTC GTC CAG ATC ATC | 24 |
| Grik1F2 | CCC CTT AGC ATA ACG ACC AG | 20 | 333 bp |
| Grik1R2 | GGC ACG AGA CAG ACA CTG AG | 20 |

Primers used for sex determination

| Primer Name | Sequence (5’-3’) | Nt | Product | Ta |
| --- | --- | --- | --- | --- |
| Sry_mm-F | GTG AGA GGC ACA AGT TGG C | 19 | 283 bp | 55 °C |
| Sry_mm-R | GTG ATG GCA TGT GGG TTC CT | 20 |
| oIMR0015-tcrd | CAA ATG TTG CTT GTC TGG TG | 20 | 200 bp |
| oIMR0016-tcrd | GTC AGT CGA GTG CAC AGT TT | 20 |
